# Occupational and psychosocial correlates of sleep disturbance among Chinese expatriate employees in Iraq’s Maysan oilfields: a cross-sectional study using regression and network analysis

**DOI:** 10.3389/fpsyt.2026.1845239

**Published:** 2026-06-10

**Authors:** Ziming Liu, Meihui Li, Chuanjiang Yang, Chun’e Zhang, Gonglu Gu, Yiran Zhang, Hongye Liu, Jing Du, Yi Fu, Shun Han, Qingwei Li

**Affiliations:** 1 Tongji University School of Medicine, Shanghai, China; 2 Department of Psychiatry, Shanghai Yangpu District Mental Health Center, Shanghai, China; 3 Office of Hospital Trade Union Committee, Shanghai Tongji Hospital, Shanghai, China; 4 CNOOC Iraq Limited, Beijing, China; 5 HYGEA Health Management (Shanghai) Co., Ltd, Shanghai, China; 6 Department of Psychiatry, Shanghai Tongji Hospital, Shanghai, China; 7 Baoshan District Integrated Traditional Chinese and Western Medicine Hospital, Shanghai, China; 8 Shanghai Mental Health Center, Shanghai, China

**Keywords:** burnout, expatriate employees, Iraq, China, occupational stress, sleep disturbance, Pittsburgh Sleep Quality Index (PSQI), psychosocial factors

## Abstract

**Introduction:**

Chinese expatriate oilfield employees working in Iraq are exposed to multiple occupational and psychosocial stressors, yet evidence on sleep disturbance and its correlates in this workforce remains limited.

**Methods:**

A cross-sectional survey was conducted among 917 employees. 826 (90.1%) were included after excluding incomplete or invalid responses. Sleep quality and related concern was assessed using the Pittsburgh Sleep Quality Index (PSQI) and the second item of the General Health Questionnaire-12 (GHQ-12). Depression, anxiety, occupational stress, burnout dimensions, Iraq-specific environmental stress, and perceived social support were measured using validated instruments and context-specific questionnaires. Multivariable linear regression and regularized partial correlation network analyses were performed.

**Results:**

The median global PSQI score was 3 (IQR 1–6). Sleep disturbance prevalence was 11.9% (95% CI 9.7%–14.3%) using PSQI > 7 and 25.2% (95% CI 22.3%–28.3%) using PSQI > 5, while 61.3% (95% CI 57.9%–64.7%) reported sleep-related concern on the GHQ-12. In the fully adjusted regression model (adjusted R^2^ = 0.522), poorer sleep quality was independently associated with higher educational attainment (high school or junior college: β = 0.68, p = 0.012; bachelor degree or above: β = 0.89, p = 0.011), more depressive symptoms (β = 0.25, p < 0.001), higher occupational stress (β = 0.03, p = 0.022), greater emotional exhaustion (β = 0.90, p < 0.001), and greater environmental stress (β = 0.57, p = 0.002). In the global network, PSQI had direct edges with depressive symptoms, anxiety symptoms, emotional exhaustion, and environmental stress and showed moderate centrality (strength = 0.69; expected influence = 0.61), with good stability of strength (CS-coefficient = 0.75). In the component network, subjective sleep quality and daytime dysfunction had the highest centrality among PSQI domains.

**Conclusion:**

Although overall self-reported sleep quality was relatively good among Chinese expatriate employees in Iraq, subclinical sleep problems and sleep-related concern were common. Poorer sleep quality was more consistently linked to depressive symptoms, work-related stress and exhaustion, and environmental stressors, while subjective sleep quality and daytime dysfunction emerged as key sleep domains. These findings may help inform priorities for improving sleep health in this expatriate oilfield workforce.

## 1 Introduction

Chinese expatriate employees, defined as Chinese workers dispatched overseas for job assignments, represent an increasingly important yet understudied population in international occupational health research (1–3). In 2024, Chinese enterprises dispatched 409,000 workers overseas, with nearly 600,000 Chinese workers abroad at the end of the year (4). These employees may be exposed to extreme environmental conditions, prolonged work shifts, occupational hazards, limited access to medical services, and extended separation from their families, particularly when assigned to regions marked by geopolitical conflict or post-conflict instability (1, 5–8). Such stressors may have profound implications for physical and mental health, including sleep quality (9).

Sleep disturbances, including difficulty initiating or maintaining sleep and nonrestorative sleep, are commonly regarded as indicators of psychological distress (10). In the general Chinese population, the prevalence of self-reported sleep disturbance ranges from 8.3% to 21.25% (11, 12). Evidence from the COVID-19 pandemic further suggests that contextual disruptions can worsen sleep problems (13–16). Recent data-driven research further highlights the importance of population-specific assessment of insomnia risk (17). Higher rates have also been reported in populations exposed to occupational or environmental stressors, such as migrant workers, among whom the prevalence of poor sleep quality has been estimated at up to 25.4% (18). Previous studies of migrant populations have primarily focused on domestic migrant workers, internal migrants, international migrants in host countries or refugees (19, 20). Several studies have reported that poor sleep is associated with factors such as shift work (21), low income (22), physical exhaustion (23), and psychological symptoms, particularly depression and anxiety (24–26).

However, evidence remains limited for employees dispatched by home-country employers to overseas industrial worksites. This gap is especially relevant for Chinese oilfield employees stationed in Iraq, a distinct but understudied group facing environmental and occupational conditions that may be linked to sleep-related concerns and problems. This focus responds to previous calls for greater attention to occupational groups that have received limited investigation (27). Therefore, examining sleep quality in this population contributes to existing evidence on mobile populations and provides context-specific evidence for occupational sleep health management in overseas assignments.

To address this gap, we investigated sleep quality and sleep-related concern among Chinese expatriate oilfield employees deployed in Iraq using self-report measures. Specifically, we estimated the prevalence of self-reported sleep disturbance and concern, examined occupational, psychological, and social correlates of sleep quality, and applied network analysis to characterize how global sleep quality and specific sleep components were associated with psychosocial and occupational factors. Together, these analyses aimed to clarify both the burden of sleep disturbance and the pattern of sleep-related associations in this understudied occupational population, thereby identifying factors that may inform future occupational health management.

## 2 Materials and methods

### 2.1 Study design and participants

A cross-sectional survey was conducted between April and June 2024 among Chinese expatriate oilfield employees stationed in the Maysan region of Iraq. Participants were recruited via an anonymous structured online questionnaire distributed through several companies operating in the region, including China Oilfield Services Limited (COSL), Anton Oilfield Services (AO), and China State Construction Engineering Corporation (CSCEC). Eligibility criteria were: (1) Chinese citizenship; (2) age ≥18 years; (3) current employment in the Maysan oilfield area; and (4) provision of informed consent. Participants with incomplete questionnaires or a self-reported history of major psychiatric disorders were excluded. The study protocol was approved by the Ethics Committee of Tongji Hospital, Shanghai.

### 2.2 Measures

#### 2.2.1 Demographic and occupational information

A self-designed questionnaire was used to collect sociodemographic variables, including age, gender, marital status, number of offspring, education level, smoking, drinking, and exercise habits. Occupational characteristics included type of work, shift schedule, average working hours per week, and overtime frequency.

#### 2.2.2 Sleep quality

The Chinese version of the PSQI was used to assess self-reported sleep quality over the past month. The PSQI consists of 7 components: subjective sleep quality, sleep latency, sleep duration, habitual sleep efficiency, sleep disturbances, use of sleep medication, and daytime dysfunction. Each component is scored from 0 to 3, yielding a global score ranging from 0 to 21, with higher scores indicating poorer sleep quality. A PSQI score >7 was used to define a significant sleep disturbance, based on validation in Chinese populations (28). In addition, the second item of the General Health Questionnaire-12 (29) (“Have you lost much sleep over worry?”) was also used to assess subjective sleep-related concern as an additional analysis.

#### 2.2.3 Generalized anxiety disorder-7 (GAD-7)

Anxiety symptoms were assessed using the Chinese version of the GAD-7 (30). Each of the 7 items was scored on a 4-point Likert scale ranging from 0 (not at all) to 3 (nearly every day), producing a total score from 0 to 21. The severity of anxiety symptoms was categorized as: 0–4: Minimal, 5–9: Mild, 10–14: Moderate, ≥15: Severe.

#### 2.2.4 Patient health questionnaire-9 (PHQ-9)

Depressive symptoms were measured using the Chinese version of the PHQ-9 (31) with total scores ranging from 0 to 27. The severity of depression was categorized as: 0–4: Minimal, 5–9: mild, 10–14: Moderate, ≥15: Severe.

#### 2.2.5 Occupational stress

Occupational stress was assessed using the Chinese version of 17-item Core Occupational Stress Scale (COSS-17) (32). The COSS-17 comprises four dimensions: social support, organization and reward, demand and effort, and autonomy. Each item is rated on a 5-point Likert scale from 1 (strongly disagree) to 5 (strongly agree). Items in the social support and autonomy domains were reverse-scored so that higher scores consistently indicate greater occupational stress. Item scores were summed to yield a total score ranging from 17 to 85, with higher scores indicating greater occupational stress. Consistent with prior studies, a total score ≥50 was used to indicate the presence of significant occupational stress (33).

#### 2.2.6 Burnout

Burnout was assessed via the Chinese version of the Maslach Burnout Inventory – General Survey (MBI-GS) (34), which includes three dimensions: emotional exhaustion, cynicism, and professional efficacy. Subscale scores were calculated as mean item scores, yielding a 0 to 6 range for each dimension. Professional efficacy was reverse-scored to reflect reduced professional efficacy, with higher scores indicating lower perceived efficacy. Following a commonly used criterion in Chinese studies (35), we applied dimension-specific cut points to categorize each burnout dimension. The corresponding mean-score thresholds were 4.00 for emotional exhaustion, 1.75 for cynicism, and 1.67 for reduced professional efficacy.

#### 2.2.7 Iraq-specific environmental stress

Iraq-specific environmental stress was assessed using the Iraq-Specific Occupational and Environmental Stress Scale (IOESS-10), a 10-item questionnaire developed from qualitative interviews, literature review, and field experience to capture deployment-related environmental stressors in the Iraq oilfield setting and their perceived impact on work, such as security-related concerns, cultural and travel restrictions, harsh natural and workplace environmental conditions, and cross-cultural workplace friction. Each item was rated on a 5-point Likert-type scale from 0 (no stress) to 4 (extreme stress, work nearly impossible). The IOESS-10 score was calculated as the mean of all items, with higher scores indicating greater perceived environmental stress and greater perceived work impact. In this study, the IOESS-10 showed satisfactory internal consistency (Cronbach’s α = 0.939; McDonald’s ω = 0.949), acceptable item-level psychometric performance, stability across company subgroups, and generally moderate correlations with established psychosocial and sleep-related measures in the expected directions (**Supplementary Tables 1, 3, 4**).

#### 2.2.8 Social support

Social support was assessed using a self-developed 8-item questionnaire covering instrumental and emotional support from four sources: supervisors, coworkers, family members, and friends. Each item was rated on a 4-point Likert-type scale from 1 (never) to 4 (always). Item scores were summed to yield a total score ranging from 8 to 32, with higher scores indicating stronger perceived social support. For descriptive comparisons, social support was categorized into three groups based on the sample distribution: low (≤26), moderate (27–31), and high (32). In this study, the questionnaire showed satisfactory internal consistency (Cronbach’s α = 0.909; McDonald’s ω = 0.928), acceptable item-level psychometric performance, stability across company subgroups, and generally moderate correlations with established psychosocial and sleep-related measures in the expected directions (**Supplementary Tables 2–4**).

### 2.3 Statistical analysis

#### 2.3.1 Descriptive analysis

Descriptive statistics summarized participants’ demographic, occupational, psychological, and sleep-related characteristics. Continuous variables were presented as means ± SD or medians (IQR), and categorical variables as frequencies and percentages. Normality was tested using the Shapiro–Wilk test and histograms. Between-group differences in sleep quality assessed by PSQI were examined using the Kruskal–Wallis test for continuous variables and Chi-square or Fisher’s exact tests for categorical variables.

#### 2.3.2 Multiple linear regression analysis

To identify factors associated with sleep quality, multiple linear regression was performed with PSQI score as the dependent variable and demographic, lifestyle, occupational, and psychosocial factors as independent variables. Model assumptions were checked, and unstandardized β coefficients with 95% confidence intervals were reported. Considering potential multicollinearity, generalized variance inflation factors (GVIFs) were calculated. For predictors with more than one degree of freedom, we reported the adjusted GVIF, defined as GVIF^1/(2·Df)^, to allow comparison across terms. Multicollinearity was considered negligible when adjusted GVIF values were < 2, and potentially concerning when ≥ 5.

#### 2.3.3 Network analysis

Networks were estimated with EBICglasso using Spearman correlation matrices (γ = 0.5) and implemented in R with the bootnet and qgraph packages (36, 37). Two networks were constructed (1): global PSQI together with depressive and anxiety symptoms, occupational stress, burnout dimensions (emotional exhaustion, cynicism, reduced professional efficacy), environmental stress, and social support; and (2) the seven PSQI component scores replacing the global PSQI score. In visualizations, nodes were arranged into 4 domains: sleep, psychological, occupational, external social, with edge thickness proportional to association strength and color indicating direction. Strength and expected influence were calculated, with edge-weight accuracy and centrality stability evaluated via nonparametric and case-dropping bootstrapping. Flow diagrams were generated to summarize the direct and indirect connections of each sleep variable within the network.

To assess the stability of the network findings, three supplementary analyses were conducted. First, potential node redundancy was examined using the goldbricker procedure to evaluate whether the network structure might be affected by construct overlap. Suggested reductions were reviewed together with conceptual and measurement considerations to determine whether potentially redundant nodes should be removed or retained. Second, ggmModSelect was used as an alternative Gaussian graphical model estimator to examine the robustness of the network structure. Results were compared with the primary EBICglasso networks in terms of retained edges, edge directions, and centrality rankings. Third, exploratory subgroup network comparisons were conducted to examine whether the main network patterns were consistent across potentially distinct participant subgroups, such as cumulative days abroad. Networks were estimated separately within each subgroup using the same EBICglasso procedure, and permutation-based network comparison tests were used to examine differences in overall network structure and global strength.

All analyses were performed using R version 4.5.1. A two-sided p < 0.05 was considered statistically significant.

## 3 Results

**Figure 1:**
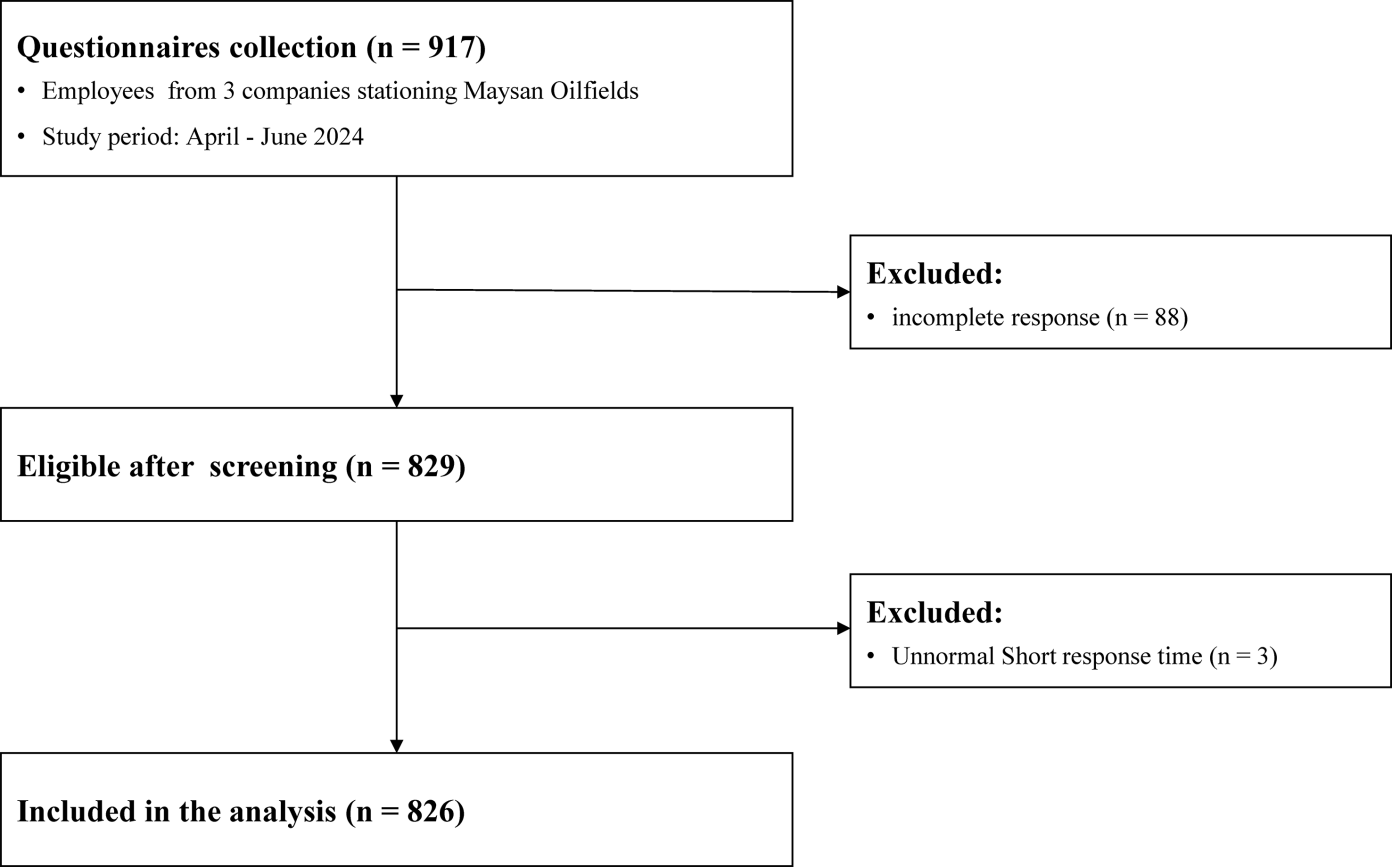
Study flow diagram. Flowchart showing the recruitment of Chinese expatriate employees in Iraq’s Maysan oilfields, the inclusion and exclusion procedures, and the final analytic sample used for the present analyses.

A total of 917 individuals participated in the survey, of whom 826 (90.1%) were included in the final analysis after excluding incomplete or invalid responses (**Figure 1**).

### 3.1 Proportion of sleep disturbance

The median global PSQI score was 3 (IQR 1–6), indicating overall sleep quality was generally adequate among included employees. A total of 11.9% (95% CI: 9.7%–14.3%) met the threshold for significant sleep disturbance (PSQI > 7). When a more inclusive criterion was applied (PSQI > 5), the proportion rose markedly to 25.2% (95% CI: 22.3%–28.3%), highlighting a considerable subclinical burden of sleep disturbance. Furthermore, 61.3% (95% CI: 57.9%–64.7%) of participants reported general health concerns related to sleep, as indicated by a positive response to the GHQ-12 sleep disturbance item, reflecting elevated subjective concern about sleep quality within overall group.

### 3.2 Subgroup differences in PSQI scores

Group comparisons of PSQI scores across demographic, lifestyle-related, occupational, and psychosocial categories are presented in **Table 1**. PSQI scores varied across several demographic characteristics, including educational attainment, marital status, and number of children, whereas no significant differences were observed by age group. Differences in PSQI scores were also observed across selected lifestyle and occupational categories, particularly smoking, alcohol consumption, overtime exposure, and cumulative days abroad. In addition, PSQI scores differed across categories of psychological symptoms, work-related stress, burnout dimensions, and perceived social support.

**Table 1:** Global PSQI score by demographic, lifestyle, occupational, and psychosocial correlates (N=826).

Variable	n	%	PSQI (median [P25, P75])	P
Overall	826		3.00 [1.00, 6.00]	
Demographic correlates				
Age				0.213
20–39 years	411	49.8%	3.00 [1.00, 6.00]	
40–60 years	415	50.2%	3.00 [1.00, 5.00]	
Education				<0.001
Middle school or below	215	26.0%	2.00 [0.00, 4.00]	
High school or junior college	331	40.1%	3.00 [2.00, 5.00]	
Bachelor degree or above	280	33.9%	4.00 [2.00, 6.00]	
Marriage				0.008
Single	74	9.0%	4.00 [2.00, 7.00]	
Married	752	91.0%	3.00 [1.00, 5.00]	
Children				<0.001
0	106	12.8%	4.00 [3.00, 7.00]	
1	260	31.5%	4.00 [2.00, 6.00]	
≥2	460	55.7%	3.00 [1.00, 5.00]	
Lifestyle correlates				
Smoking				<0.001
No	424	51.3%	3.00 [1.00, 5.00]	
Yes	402	48.7%	4.00 [2.00, 6.00]	
Drinking				<0.001
No	585	70.8%	3.00 [1.00, 5.00]	
Yes	241	29.2%	4.00 [3.00, 6.00]	
Exercise				0.437
Never	78	9.4%	3.00 [1.00, 7.00]	
Sometimes	529	64.0%	3.00 [1.00, 6.00]	
Usually	219	26.6%	3.00 [2.00, 5.00]	
Roommates				<0.001
0	261	31.6%	4.00 [2.00, 6.00]	
1	214	25.9%	4.00 [2.00, 6.00]	
≥2	351	42.5%	2.00 [1.00, 5.00]	
Occupational correlates				
Company				<0.001
COSL	380	46.0%	4.00 [2.00, 6.00]	
CSCEC	340	41.2%	2.50 [1.00, 5.00]	
AO	106	12.8%	3.00 [1.00, 5.00]	
Type of work				<0.001
Management	128	15.5%	4.00 [3.00, 6.00]	
Frontline operations	656	79.4%	3.00 [1.00, 5.00]	
Maintenance or others	42	5.1%	3.00 [1.00, 6.00]	
Years of working				<0.001
0–4 years	217	26.3%	2.00 [0.00, 5.00]	
5–9 years	148	17.9%	3.00 [1.00, 5.00]	
10–16 years	257	31.1%	4.00 [2.00, 6.00]	
≥16 years	204	24.7%	4.00 [2.00, 6.00]	
Shift				0.092
Fixed day shift	540	65.4%	3.00 [1.00, 5.00]	
Unfixed night shift	196	23.7%	3.50 [2.00, 6.00]	
Fixed night shift	90	10.9%	3.00 [2.00, 6.00]	
Cumulative days abroad				<0.001
≤30 d	225	27.2%	3.00 [2.00, 6.00]	
30–60 d	230	27.8%	3.50 [2.00, 6.00]	
60–120 d	155	18.8%	4.00 [2.00, 6.00]	
>120 d	216	26.2%	2.00 [0.00, 5.00]	
Overtime per week				<0.001
<6 h	421	51.0%	2.00 [1.00, 4.00]	
6–10 h	220	26.6%	4.00 [2.00, 6.00]	
>10 h	185	22.4%	4.00 [3.00, 7.00]	
Psychosocial correlates				
Anxiety				<0.001
Minimal	678	82.1%	3.00 [1.00, 5.00]	
Mild	133	16.1%	6.00 [5.00, 8.00]	
Moderate	13	1.6%	8.00 [7.00, 12.00]	
Severe	2	0.2%	9.50 [8.00, 11.00]	
Depression				<0.001
Minimal	588	71.2%	2.00 [1.00, 4.00]	
Mild	202	24.5%	6.00 [4.00, 8.00]	
Moderate	32	3.9%	8.00 [5.00, 10.50]	
Severe	4	0.5%	6.00 [2.00, 9.50]	
Occupational stress				<0.001
No	722	87.4%	3.00 [1.00, 5.00]	
Yes	104	12.6%	5.00 [2.00, 8.00]	
Environmental stress				<0.001
None	303	36.7%	1.00 [0.00, 3.00]	
Low	324	39.2%	4.00 [2.00, 5.00]	
High	199	24.1%	6.00 [4.00, 8.00]	
Occupational Burnout				
Emotional exhaustion				0.004
No	822	99.5%	3.00 [1.00, 5.00]	
Yes	4	0.5%	8.50 [7.00, 10.50]	
Cynicism				<0.001
No	758	91.7%	3.00 [1.00, 5.00]	
Yes	68	8.3%	7.00 [5.00, 10.00]	
Reduced professional efficacy				0.007
No	453	54.7%	3.00 [1.00, 5.00]	
Yes	373	45.3%	4.00 [1.00, 6.00]	
Social support				<0.001
Lack	217	26.3%	5.00 [3.00, 7.00]	
Moderate	219	26.5%	4.00 [2.00, 6.00]	
High	390	47.2%	2.00 [0.00, 4.00]	

Values are presented as median [P25, P75] for the global PSQI. P values were calculated using the Mann–Whitney U test for two-group comparisons and the Kruskal–Wallis test for comparisons involving three or more groups. Anxiety and depressive symptoms were assessed using the GAD-7 and PHQ-9, respectively. Occupational stress was assessed using the COSS-17. Burnout (emotional exhaustion, cynicism, and reduced professional efficacy) was assessed using the MBI–GS. Environmental stress and social support were measured using structured questionnaires. COSL, China Oilfield Services Limited; CSCEC, China State Construction Engineering Corporation; AO, Anton Oilfield Services. Bold values indicate category headings for groups of correlates.

### 3.3 Regression analysis

Regression results are shown in **Table 2**. As covariates were added, model fit improved with adjusted R^2^ rising from 0.076 in Model 1 to 0.145 in Model 2, and 0.522 in Model 3. In the fully adjusted model, educational attainment remained a stable correlate of poorer sleep quality. Relative to middle school or below, PSQI was higher among participants with high school/junior college (β = 0.68, p = 0.012) and among those with a bachelor’s degree or above (β = 0.89, p = 0.011), while age, marital status, and number of children showed no independent association. Several lifestyle and work-pattern variables showed associations with PSQI in Model 2, but these were largely attenuated in Model 3. After full adjustment, psychological symptoms and work-related burden measures remained the main correlates of PSQI. Depressive symptom severity was associated with poorer sleep quality (β = 0.25, p < 0.001), whereas anxiety symptoms were not statistically significant (β = 0.12, p = 0.052). Occupational stress remained independently associated with higher PSQI (β = 0.03, p = 0.022). For burnout dimensions, emotional exhaustion showed a strong positive association with PSQI (β = 0.91, p < 0.001), while cynicism was inversely associated (β = −0.53, p = 0.021), reduced professional efficacy was not associated. Specific environmental stress was also associated with poorer sleep quality (β = 0.57, p = 0.002), whereas perceived social support was not associated. Several work-pattern and lifestyle related indicators such as shift pattern, overtime, drinking and smoking were not independently associated with PSQI after full adjustment. By contrast, occasional exercise and cumulative days abroad >120 days remained significantly associated with lower PSQI. In the final model, the maximum adjusted GVIF was 1.919 (for PHQ-9), indicating multicollinearity is acceptable (**Supplementary Materials Supplementary Table 5**).

**Table 2:** Multiple linear regression analysis of factors associated with PSQI scores (N=826).

Predictor	Model 1		Model 2		Model 3	
	β [95% CI]	P	β [95% CI]	P	β [95% CI]	P
Age per year increase	0.03 [0.00, 0.06]	0.074	0.01 [-0.03, 0.04]	0.768	-0.01 [-0.03, 0.02]	0.729
Education (Ref: Middle school or below)	0		0		0	
High school or junior college	1.27 [0.78, 1.77]	<0.001	1.09 [0.35, 1.82]	0.004	0.68 [0.15, 1.20]	0.012
Bachelor degree or above	1.83 [1.28, 2.38]	<0.001	1.43 [0.55, 2.30]	0.001	0.89 [0.20, 1.57]	0.011
Marriage (Ref: Single)	0		0		0	
Married	-0.23 [-1.08, 0.63]	0.599	-0.27 [-1.13, 0.59]	0.537	-0.18 [-0.82, 0.46]	0.589
Children (Ref: 0)	0		0		0	
1	-0.44 [-1.28, 0.40]	0.300	-0.48 [-1.33, 0.36]	0.263	0.03 [-0.58, 0.64]	0.929
≥2	-1.01 [-1.83, -0.19]	0.015	-0.99 [-1.80, -0.18]	0.017	-0.16 [-0.76, 0.44]	0.594
Smoking (Ref: No)			0		0	
Yes			0.35 [-0.06, 0.76]	0.095	-0.09 [-0.41, 0.23]	0.601
Drinking (Ref: No)			0		0	
Yes			0.58 [0.13, 1.03]	0.011	0.16 [-0.18, 0.50]	0.345
Exercise (Ref: never)			0		0	
Sometimes			-0.97 [-1.72, -0.22]	0.011	-0.60 [-1.18, -0.01]	0.047
Usually			-1.40 [-2.25, -0.56]	0.001	-0.36 [-1.02, 0.31]	0.295
Roommates (Ref: 0)			0		0	
1			-0.24 [-0.78, 0.29]	0.374	-0.20 [-0.61, 0.21]	0.337
≥2			-0.68 [-1.41, 0.06]	0.072	-0.26 [-0.82, 0.29]	0.352
Company (Ref: COSL)			0		0	
CSCEC			1.19 [0.41, 1.97]	0.003	0.01 [-0.56, 0.59]	0.960
AO			0.19 [-0.69, 1.08]	0.669	-0.27 [-0.86, 0.33]	0.381
Type of work (Ref: Management)			0		0	
Frontline operations			0.10 [-0.54, 0.73]	0.765	-0.07 [-0.53, 0.39]	0.777
Maintenance or others			-0.00 [-1.22, 1.22]	0.999	0.03 [-0.86, 0.92]	0.950
Years of working (Ref: 0–4 years)			0		0	
5–9 years			0.03 [-0.56, 0.63]	0.913	-0.02 [-0.47, 0.42]	0.919
10–16 years			0.74 [0.17, 1.30]	0.010	0.10 [-0.32, 0.52]	0.635
≥16 years			0.88 [0.21, 1.55]	0.010	0.51 [0.01, 1.01]	0.046
Shift (Ref: Fixed day shift)			0		0	
Unfixed night shift			-0.09 [-0.61, 0.43]	0.738	-0.18 [-0.58, 0.22]	0.380
Fixed night shift			0.27 [-0.63, 1.17]	0.556	0.23 [-0.40, 0.87]	0.466
Cumulative days abroad (Ref: ≤30 d)			0		0	
30–60 d			0.17 [-0.34, 0.67]	0.517	-0.12 [-0.50, 0.27]	0.551
60–120 d			0.38 [-0.22, 0.99]	0.215	0.32 [-0.12, 0.76]	0.153
>120 d			-0.17 [-0.83, 0.49]	0.612	-0.57 [-1.05, -0.08]	0.023
Overtime per week (Ref: <6 h)			0		0	
6–10 h			0.61 [0.14, 1.08]	0.011	0.17 [-0.20, 0.54]	0.357
>10 h			1.06 [0.49, 1.63]	<0.001	0.33 [-0.12, 0.79]	0.147
PHQ-9					0.25 [0.15, 0.35]	<0.001
GAD-7					0.12 [-0.00, 0.24]	0.054
COSS-17					0.03 [0.00, 0.05]	0.022
Emotional exhaustion					0.90 [0.53, 1.27]	<0.001
Cynicism					-0.53 [-0.97, -0.08]	0.021
Reduced professional efficacy					0.02 [-0.08, 0.11]	0.757
IOESS					0.57 [0.21, 0.92]	0.002
Social support					-0.13 [-0.52, 0.25]	0.487

Model fit: Model 1 (R^2^ = 0.0829, adjusted R^2^ = 0.0761); Model 2 (R^2^ = 0.1592, adjusted R^2^ = 0.1340); Model 3 (R^2^ = 0.5379, adjusted R^2^ = 0.5193). Unstandardized β coefficients with 95% confidence intervals are shown; p-values and confidence intervals were computed using HC3 heteroscedasticity-consistent standard errors. COSL, China Oilfield Services Limited; CSCEC, China State Construction Engineering Corporation; AO, Anton Oilfield Services.

### 3.4 Network analysis

#### 3.4.1 Network structure including global sleep quality

**Figure 2:**
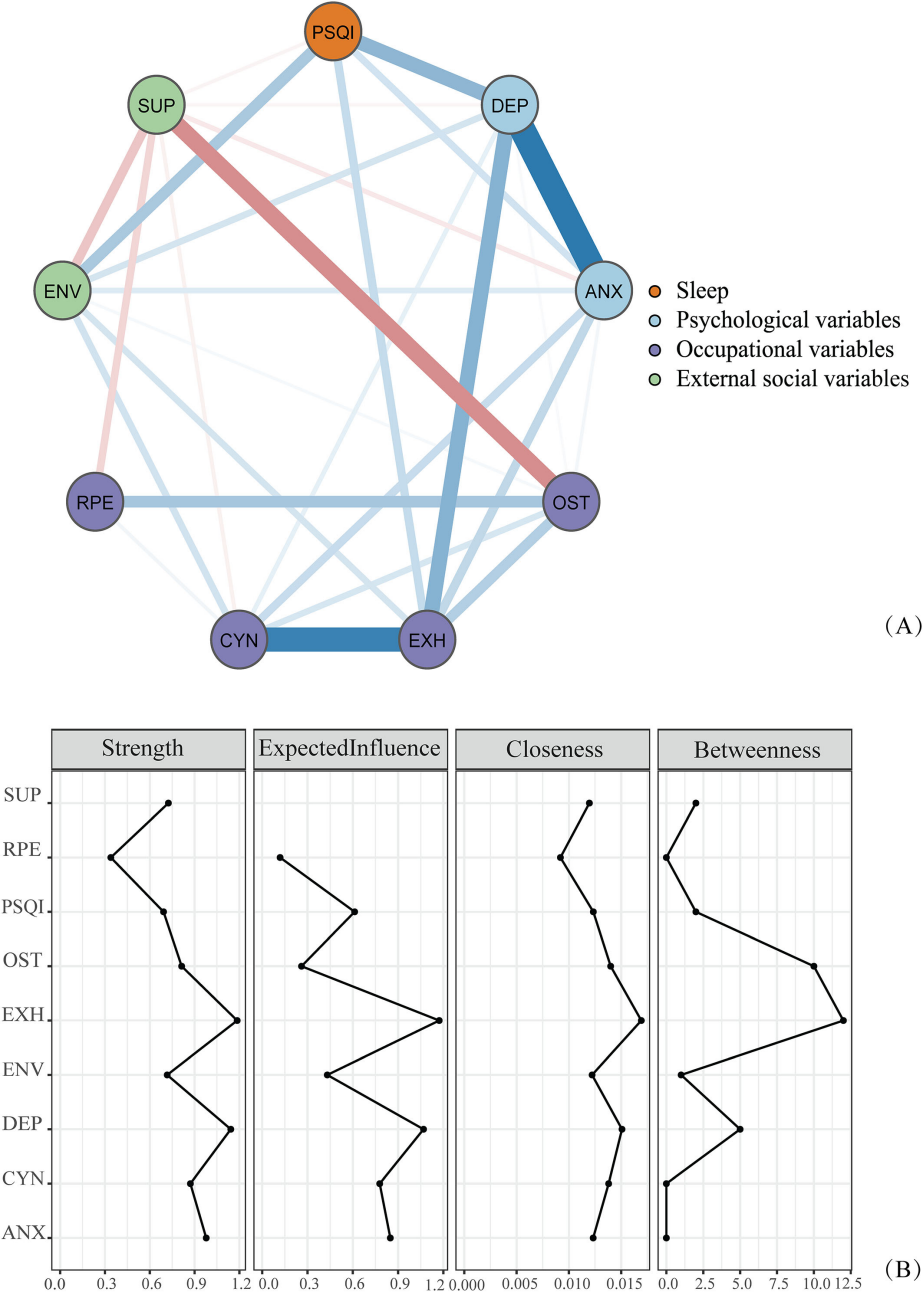
Network structure and centrality of global sleep disturbance. **(A)** Network structure including global sleep disturbance and psychosocial variables. PSQI, global sleep disturbance; DEP, depressive symptoms; ANX, anxiety symptoms; OST, occupational stress; EXH, emotional exhaustion; CYN, cynicism; RPE, reduced professional efficacy; ENV, environmental stress; SUP, social support. Edges represent regularized partial correlations estimated using the EBICglasso method. Blue edges indicate positive associations, whereas red edges indicate negative associations. Edge thickness reflects the magnitude of the association after regularization. Nodes are colored according to conceptual domains, with orange node representing the global PSQI, light blue representing psychological variables, purple nodes representing occupation-related dimensions and green nodes representing external social-related variables. **(B)** Centrality indices, including strength, expected influence, closeness and betweenness of each node. Higher values indicate greater importance of a node within the multivariate network structure.

In the network including global sleep quality (**Figure 2A**; **Supplementary Materials Supplementary Table 6**), the PSQI global score showed positive regularized partial correlations with depressive symptoms, anxiety symptoms, emotional exhaustion, and environmental stress after conditioning on the remaining nodes. Centrality analysis (**Figure 2B**; **Supplementary Materials Supplementary Table 7**) showed that emotional exhaustion and depressive symptoms exhibited relatively high strength and expected influence within the network. The global PSQI score showed moderate centrality in the overall network (strength = 0.69; expected influence = 0.61). Nonparametric bootstrap confidence intervals for edge weights suggested acceptable accuracy of the estimated edges (**Supplementary Materials Supplementary Figure 1**). Case-dropping bootstrap indicated good stability of strength centrality (CS-coefficient = 0.75; **Supplementary Figure 2**). Overall, global sleep quality was not the most central node but remained moderately central and closely connected with psychological, burnout-related, and environmental stress variables in this multidimensional network.

#### 3.4.2 Flow analysis centered on global sleep quality

**Figure 3:**
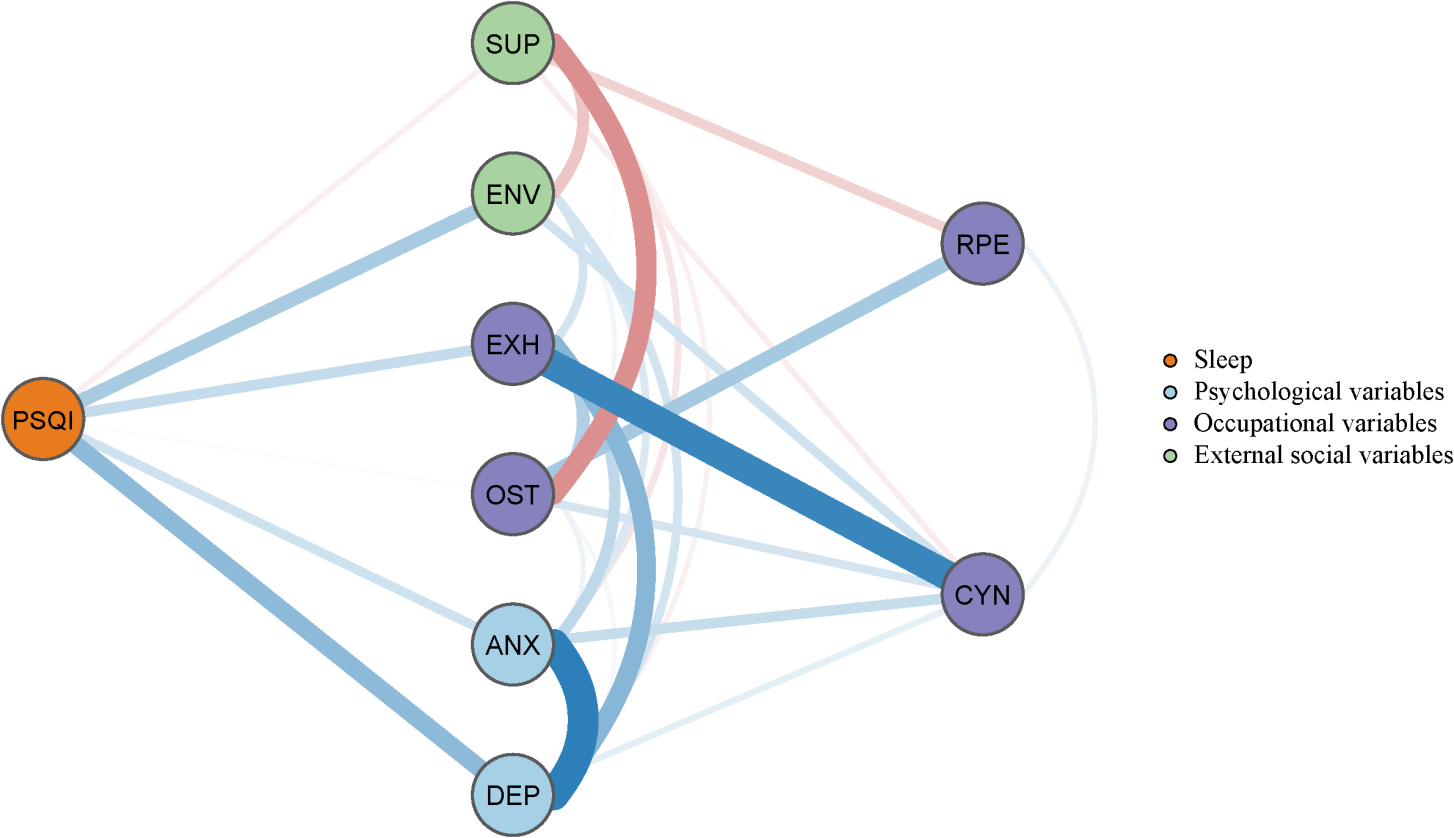
Flow network of global sleep disturbance. The flow network illustrates direct and indirect associations between global PSQI and psychosocial and occupational variables. PSQI, global sleep disturbance; DEP, depressive symptoms; ANX, anxiety symptoms; OST, occupational stress; EXH, emotional exhaustion; CYN, cynicism; RPE, reduced professional efficacy; ENV, environmental stress; SUP, social support. Edges represent regularized partial correlations retained in the network. Blue edges indicate positive associations and red edges indicate negative associations, with edge thickness corresponding to association strength.

In the flow network with the global sleep quality as the focal node (**Figure 3**), depressive symptoms, environmental stress, and emotional exhaustion were directly connected to global sleep disturbance. Anxiety symptoms and social support also appeared as direct neighbors, with the edge to social support being weakly negative. Occupational stress had no direct edge to global sleep quality in this flow network.

#### 3.4.3 Network including PSQI components

**Figure 4:**
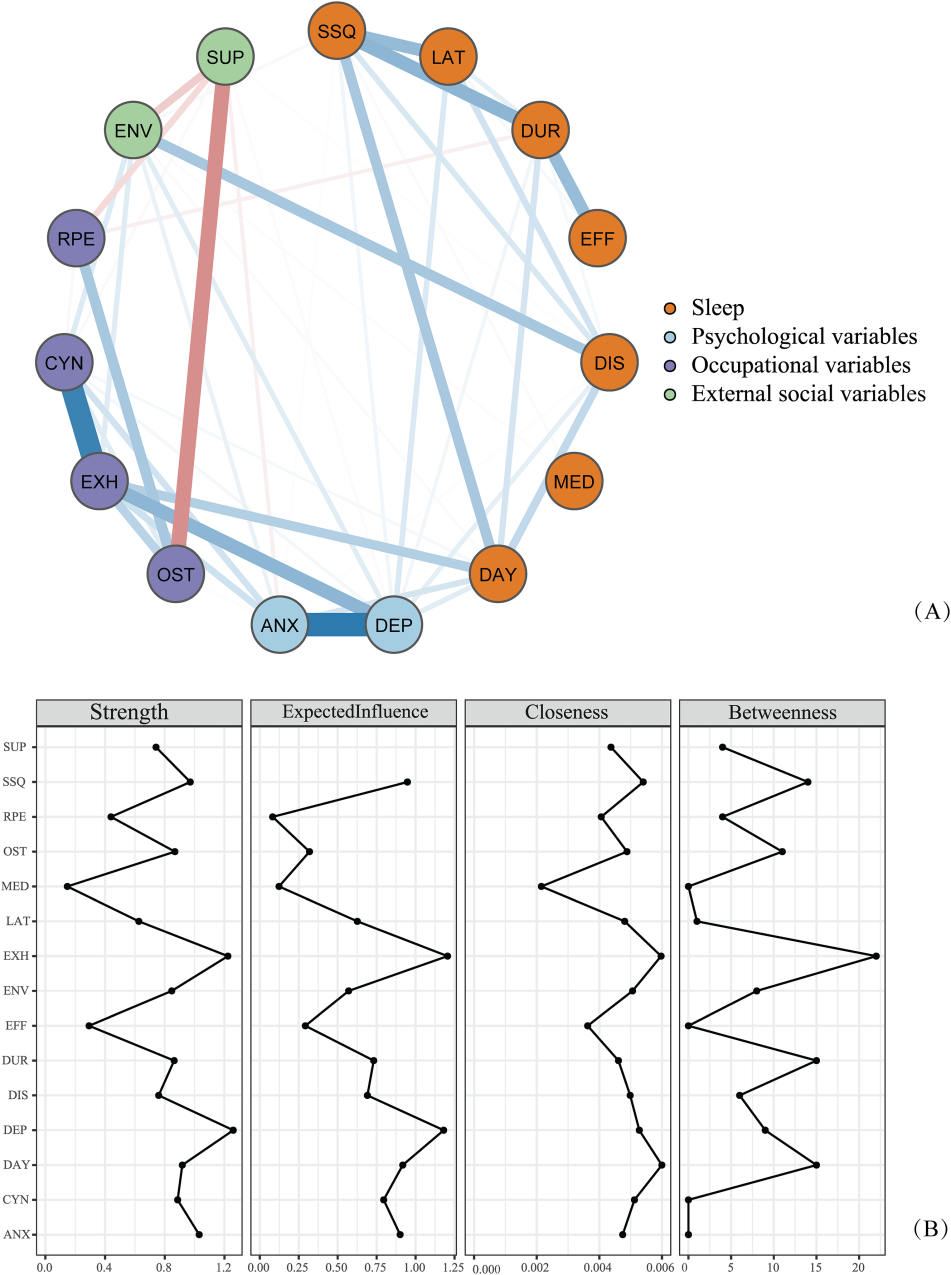
Network structure and centrality of PSQI components. **(A)** Network structure including PSQI components and psychosocial variables. Nodes represent the seven PSQI components and psychosocial variables. SSQ, subjective sleep quality; LAT, sleep latency; DUR, sleep duration; EFF, habitual sleep efficiency; DIS, sleep disturbances; MED, sleep medication use; DAY, daytime dysfunction; DEP, depressive symptoms; ANX, anxiety symptoms; OST, occupational stress; EXH, emotional exhaustion; CYN, cynicism; RPE, reduced professional efficacy; ENV, environmental stress; SUP, social support. Nodes are colored according to conceptual domains, with orange node representing the global PSQI, light blue representing psychological variables, purple nodes representing occupation-related dimensions and green nodes representing external social-related variables. Edges represent regularized partial correlations, with blue edges indicating positive associations and red edges indicating negative associations. Edge thickness reflects the magnitude of the association. **(B)** Centrality indices of the PSQI component network. Centrality measures including strength, expected influence, closeness and betweenness are displayed for all nodes in the network, indicating the relative importance of individual sleep components and psychosocial variables within the network.

**Figure 4A** presents the component-level network including the seven PSQI domains and psychosocial variables (**Supplementary Materials Supplementary Table 8**). Within the sleep domain, the strongest within-sleep edges were observed between sleep duration and subjective sleep quality (edge weight = 0.24), sleep latency and subjective sleep quality (edge weight = 0.23), and habitual sleep efficiency and sleep duration (edge weight = 0.22). Daytime dysfunction showed direct edges with subjective sleep quality (edge weight = 0.19), sleep disturbances (edge weight = 0.14), and sleep duration (edge weight = 0.11). Centrality analysis (**Figure 4B**; **Supplementary Materials Supplementary Table 9**) showed that subjective sleep quality and daytime dysfunction had higher strength and expected influence than the other PSQI components. Bootstrap confidence intervals for edge weights suggested acceptable accuracy (**Supplementary Materials Supplementary Figure 3**). Case-dropping bootstrap indicated good stability of strength centrality (CS-coefficient = 0.75; **Supplementary Materials Supplementary Figure 4**). Overall, subjective sleep quality and daytime dysfunction were the most central PSQI components in this multidimensional network.

#### 3.4.4 Flow analysis of individual sleep components

**Figure 5:**
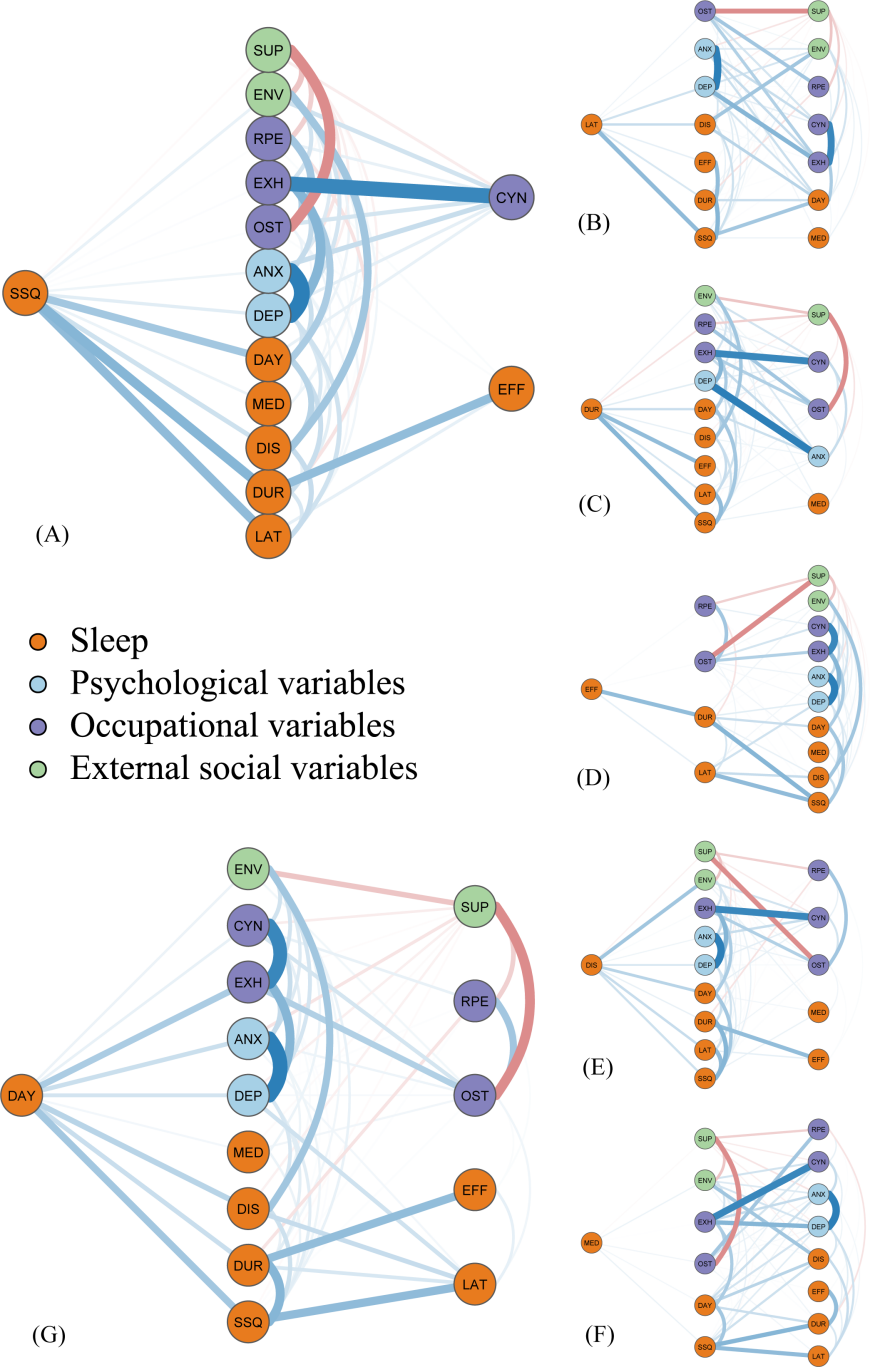
Flow networks of individual PSQI domains. **(A–G)** Each graph presents a flow network with individual PSQI component as the focal node: **(A)** SSQ, subjective sleep quality; **(B)** LAT, sleep latency; **(C)** DUR, sleep duration; **(D)** EFF, habitual sleep efficiency; **(E)** DIS, sleep disturbances; **(F)** MED, sleep medication use and **(G)** DAY, daytime dysfunction. Edges represent regularized partial correlations, with blue edges indicating positive associations and red edges indicating negative associations. Edge thickness corresponds to the magnitude of associations. The flow networks illustrate component-specific patterns of direct and indirect connections with psychosocial and occupational variables.

Flow networks for individual PSQI components (**Figures 5A–G**) showed that subjective sleep quality and daytime dysfunction had more cross-domain edges with psychological, burnout-related, and occupational stress variables than the other PSQI components. The remaining PSQI components showed fewer direct cross-domain edges and were more closely connected within the sleep-domain structure.

#### 3.4.5 Sensitivity and subgroup network analyses

Node redundancy analysis indicated one potential redundant pair in the global PSQI network, involving global PSQI and specific environmental stress, whereas no suggested reductions were identified in the PSQI component network (**Supplementary Table 10**). Given their distinct measurement content, both nodes were retained in the primary network models.

The ggmModSelect sensitivity analysis yielded sparser networks than the primary EBICglasso models. In the global PSQI network, 17 of 30 edges were retained, corresponding to an edge retention rate of 56.7%. In the PSQI component network, 29 of 68 edges were retained, corresponding to an edge retention rate of 42.6% (**Supplementary Figures 5, 6**; **Supplementary Tables 11–13**). All retained edges showed the same direction of association across estimators. In the global PSQI network, the PSQI edges with depressive symptoms, emotional exhaustion, and environmental stress were retained, whereas the weaker PSQI–anxiety edge was not retained. Overall, the sensitivity analysis supported the main sleep-related associations.

Subgroup network comparison showed no significant differences between the ≤120-day and >120-day cumulative-stay groups in overall network structure or global strength, for either the global PSQI network (M = 0.181, p = 0.253; S = 0.162, p = 0.432) or the PSQI component network (M = 0.189, p = 0.207; S = 0.445, p = 0.149; **Supplementary Figures 7, 8**). These findings suggested that both the global sleep quality network and the component-level sleep network were broadly consistent across cumulative overseas-stay groups.

## 4 Discussion

In this cross-sectional study among Chinese expatriate oilfield employees, overall self-reported sleep quality was relatively good, yet sleep-related problems were still common. About one in eight participants met the clinical PSQI threshold for poor sleep, about one in four reported at least mild sleep disturbance, and a majority reported sleep-related health concern, indicating a sizeable subclinical burden. In multivariable models, poorer sleep was most consistently associated with depressive symptoms, emotional exhaustion, occupational stress, and environmental stress. Anxiety symptoms and perceived social support did not show independent associations after accounting for these co-occurring factors. The network results aligned with this pattern and further indicated that subjective sleep quality and daytime dysfunction were the PSQI components most tightly linked to psychosocial and occupational strain.

### 4.1 Sleep-related burden beyond clinical thresholds

Although the prevalence of clinically defined sleep disturbance was relatively low among the included employees, the much higher proportions of subclinical sleep disturbance and sleep-related concern suggest a substantial subclinical burden of sleep problems. Previous studies reported that environmental stress exposure may amplify sleep-related concerns among expatriate and migrant workers (19, 20), yet the observed proportion of clinical sleep disturbance is markedly below estimates from the general Chinese adult (11, 38), elderly (39) and specific occupational groups (11, 40, 41). In particular, compared with a recent studies of workers stationed in similar environments in Shaanxi and Hainan, China (42, 43), the present study found a much lower prevalence of clinically defined self-reported sleep disturbance. Even so, sleep-related concern remained strikingly common and was more frequent than that reported in some other high-stress occupational settings (29, 44). Moreover, compared with refugee and local resident samples from the Middle Eastern region (16, 45), the prevalence of self-reported poor sleep in the present sample was also relatively low. Nevertheless, the high proportion of subclinical sleep disturbance and sleep-related concern indicates that sleep problems remained relevant in this expatriate workforce.

The discrepancy between PSQI >7, PSQI >5, and sleep-related concern may reflect cutoff selection and occupational selection. Consistent with our findings, recent empirical research in migrant worker populations has shown that poor sleep quality can be common when a more sensitive PSQI cutoff is used (46). In the present study, the stricter threshold appeared to identify only the more severe part of self-reported sleep problems. The relatively low prevalence at PSQI >7 may also reflect selection into overseas deployment, as employees who accept expatriate assignments may represent a comparatively healthier or more resilient subgroup than the general population (47). More broadly, recent evidence suggests that the optimal PSQI cutoff can vary across populations and according to the intended use of the instrument, underscoring the need for population-specific validation rather than direct transfer of a single threshold across settings (48–50). In this context, a stricter cutoff may not fully capture the broader burden of sleep problems in expatriate employees, although this should be formally tested in future validation studies.

Cultural and occupational reporting contexts may also be relevant when considering these prevalence differences. Recent cross-cultural evidence suggests that healthy sleep duration varies across cultures, indicating that sleep norms and judgments about adequate or problematic sleep may not be fully captured by a single universal standard (51). For Chinese employees stationed overseas, the appraisal of whether sleep difficulties reach a health-concerning level may therefore be shaped by both Chinese cultural sleep norms and the expectations of a deployed occupational setting. In broader research on mobile and culturally diverse populations, social identity-related stressors, including racial or ethnic discrimination, have been linked to sleep difficulties, while ethnic identity may buffer some of these associations (52–54). Compared with refugees or involuntary migrants, employer-dispatched expatriate workers may have stronger occupational and cultural identity, which could partly buffer stress related to cross-cultural adaptation or perceived discrimination. This may provide one possible context for the relatively low prevalence of clinical-threshold self-reported sleep disturbance observed in this sample. Overall, the low prevalence at PSQI >7, together with the marked increase at PSQI >5 and the frequent sleep-related concern, suggests that sleep-related burden in this expatriate workforce was concentrated largely at subclinical levels and may be underestimated when only the stricter threshold is used.

### 4.2 Independent correlates of poorer sleep quality

After multivariable adjustment, psychological factors and work-related burden showed the strongest associations with sleep quality. Depressive symptom severity was consistently associated with higher PSQI scores, whereas the association for anxiety symptoms was attenuated and no longer statistically significant in the fully adjusted model. This pattern is consistent with previous studies showing that depressive symptoms are closely linked to poorer sleep quality across both community and occupational samples (55–57). Cross-national evidence has also suggested close interrelations among work-related stress, sleep disturbance, and depressive symptoms (58). Recent evidence further suggests that, relative to anxiety, depression may show a stronger association with several clinically salient sleep domains, particularly subjective sleep quality, daytime dysfunction, and sleep disturbances (59). In our model, the attenuation of anxiety after full adjustment may therefore reflect overlap with depressive symptoms and other co-occurring stress-related burdens, rather than the absence of any relationship with sleep.

Among work-related burden variables, occupational stress and emotional exhaustion were independently associated with poorer sleep quality, suggesting a close link between job-related strain and sleep problems in this setting. Previous studies have consistently linked emotional exhaustion with poorer sleep, particularly in high-stress occupational environments (60, 61), and a recent large study of oilfield workers similarly reported dose–response associations between occupational burnout and poor sleep quality (42). By contrast, cynicism showed an inverse association after full adjustment. Given that cynicism reflects psychological distance or negative attitudes toward work and overlaps conceptually with other burnout dimensions, this inverse coefficient is unlikely to indicate a protective association and may reflect suppression after adjustment for emotional exhaustion and reduced professional efficacy.

Contextual stress and support factors showed a less symmetrical pattern. Environmental stress remained independently associated with poorer sleep quality, consistent with the evidence that ongoing environmental uncertainty and perceived threat can make it harder to sleep, even after accounting for mood symptoms and occupational stress (19, 20). By contrast, perceived social support was not independently associated with sleep quality in the fully adjusted model. This does not necessarily indicate that support is unimportant; rather, its sleep-related role in this setting may be weaker than that of co-occurring stress and affective burden, or may operate more indirectly as a buffering resource. Recent occupational evidence supports this interpretation by showing that low workplace social support is associated with insomnia in a dose–response manner, with part of this relationship mediated through job satisfaction rather than expressed solely as a direct effect (62).

Several lifestyle and work-pattern indicators, including smoking, alcohol consumption, overtime, and shift pattern, were associated with PSQI in unadjusted analyses but were not independently related to sleep quality after full adjustment. Large cohort and population studies consistently report worse sleep with long hours and atypical schedules, and evidence from oil-industry shift workers similarly highlights short sleep and impaired sleep quality around rotations, early start times, and overtime (63–65). The attenuation of these associations after full adjustment suggests that, in this setting, the effects of lifestyle and work-pattern factors on sleep may be partly accounted for by more proximal psychological stressors, particularly depressive symptoms, perceived stress, and emotional exhaustion. By contrast, Higher educational attainment remained associated with poorer sleep quality after adjustment, which differs from some population-based findings linking higher education to better sleep (38, 66). Evidence on the education–sleep relationship is mixed. A recent population-based study reported lower odds of poor sleep quality among adults with higher education (67), whereas other evidence suggests that education may not be a stable stand-alone predictor and may be better understood within broader socioeconomic and occupational contexts (68, 69). The reasons behind this finding are not yet clear, but employees with higher education may represent a subgroup whose sleep problems warrant closer attention in this setting.

### 4.3 Sleep quality in a stress-related context: key domains and cross-domain links

The network analyses provided complementary insight into how sleep disturbance was situated within the broader pattern of occupational and psychosocial strain. In the present network, global PSQI was directly connected with depressive symptoms, anxiety symptoms, emotional exhaustion, and environmental stress, consistent with prior evidence linking sleep disturbance with affective and stress-related processes (70, 71). Depressive symptoms and emotional exhaustion also showed comparatively high centrality, indicating that these variables occupied structurally prominent positions within the overall network. This finding aligns with established burnout models that identify emotional exhaustion as a key stress-response component of burnout (72), as well as with recent network studies showing that sleep, depression, anxiety, and burnout-related symptoms often form closely interconnected structures rather than independent domains (73–79). The PSQI-centered flow display further showed that depressive symptoms, anxiety symptoms, emotional exhaustion, and environmental stress were closer to sleep than other variables in the estimated network. These findings suggest that, within the broader pattern of work-related and psychosocial burden, sleep disturbance was more closely aligned with affective burden, emotional exhaustion, and environmental stress. The sleep quality–anxiety edge observed in the primary network was not retained in the sensitivity analysis, suggesting that this direct association was less stable than the links of sleep with depressive symptoms, emotional exhaustion, and environmental stress. This is consistent with the multivariable regression results, in which anxiety symptoms did not show an independent association after accounting for co-occurring depressive and occupational factors.

At the component level, the central position of subjective sleep quality and daytime dysfunction suggests that perceived sleep quality and daytime consequences may be the most informative domains for understanding sleep-related burden in this deployment setting. This is relevant for expatriate oilfield employees because sleep problems may be most noticeable when experienced as nonrestorative sleep or impaired daytime functioning. Cultural differences in sleep duration and sleep timing may also shape how employees judge sleep adequacy and daytime impact (51, 80). Prior studies similarly identified subjective sleep quality and daytime impairment as clinically relevant aspects of sleep disturbance (71, 81), and recent component-network studies have shown that these domains may occupy central positions or serve as cross-domain linking symptoms in specific populations (82–84). Together, these findings suggest that sleep disturbance in this expatriate workforce was closely embedded in a stress-related context, while perceived sleep quality and daytime functioning may provide practical domains for recognizing sleep-related burden.

### 4.4 Implications and limitations

Our findings have several practical implications for occupational sleep health management in expatriate settings. First, sleep assessment in this workforce may need to place greater emphasis on identifying mild-to-moderate sleep-related burden, with attention not only to clinical thresholds but also to the PSQI domains most closely linked to perceived burden and daytime impact—this may better capture employees with meaningful sleep-related burden and functional impact. Second, Evaluation of sleep complaints may benefit from considering co-occurring depressive symptoms, emotional exhaustion, occupational stress, and environmental stress, rather than focusing only on work schedules. Third, the persistent association between environmental stress and sleep quality highlights the importance of organizational measures that improve rest conditions and provide accessible and confidential psychological support for employees deployed in high-stress environments.

Several limitations should be noted. First, the cross-sectional design limits conclusions about causality or directionality between sleep disturbance and the included variables. Second, all measures were based on self-reported questionnaires and objective sleep assessments such as polysomnography or actigraphy were not included. Therefore, recall bias, reporting bias, and common method variance cannot be excluded. Moreover, the present study could not further characterize objective sleep architecture or identify potential underlying sleep disorder phenotypes. Third, participants were recruited from a single overseas worksite in the Maysan region of Iraq, and non-response or exclusion of incomplete questionnaires may have introduced selection bias. The generalizability of the findings to other expatriate workforces or occupational and geopolitical contexts therefore requires further validation. Finally, although the Iraq-Specific Occupational and Environmental Stress Scale and the social support questionnaire were developed for this occupational context and showed acceptable psychometric performance, further independent large-sample validation is needed to compare them with established instruments and extend their applicability to other similar occupational settings. Future longitudinal, multi-site studies incorporating objective sleep monitoring and externally validated measures are needed to confirm these findings and clarify the temporal relationships between sleep disturbance and psychosocial or occupational factors.

## 5 Conclusion

Overall self-reported sleep quality was relatively good among Chinese expatriate employees in Iraq, but subclinical sleep disturbance was common, and most participants reported concern about their sleep. Poorer self-reported sleep quality was more consistently associated with psychological, occupational, and environmental strain, particularly depressive symptoms, occupational stress, emotional exhaustion, and specific environmental stressors than with lifestyle or work-pattern characteristic. Sleep quality appeared to be embedded in a broader pattern of psychological, occupational, and environmental strain, while subjective sleep quality and daytime dysfunction emerged as especially important sleep dimensions. Together, these findings highlight the importance of recognizing the subclinical burden of sleep and specific sleep dimensions, and suggest that sleep quality in this expatriate oilfield workforce may be better understood within an integrated framework spanning sleep, work-related burden especially occupational stress, exhaustion, psychological strain, and environmental stress, which may also help inform practical priorities to improve employees’ sleep health.

## Data Availability

The de-identified raw data supporting the conclusions of this article will be made available by the corresponding author upon reasonable request, without undue reservation, subject to participant confidentiality requirements and permission from the relevant ethics committee and participating organization. Requests to access the datasets should be directed to Qingwei Li, lianocd@tongji.edu.cn.
