# Supplementary material for: Occupational and psychosocial correlates of sleep disturbance among Chinese expatriate employees in Iraq’s Maysan oilfields: a cross-sectional study using regression and network analysis
Source: Front Psychiatry. 2026 Jun 10;17:1845239. doi: 10.3389/fpsyt.2026.1845239 (PMC13291141; doi:10.3389/fpsyt.2026.1845239)
Supplement: Supplementary file 1 [file SupplementaryFile1.docx]

Supplementary Material

# Supplementary Figures and Tables

## Supplementary Figures

**Supplementary Figure S1. Bootstrap edge-weight accuracy for the global sleep disturbance network**

**Supplementary Figure S2. Case-dropping bootstrap stability of strength centrality for the global sleep disturbance network**

**Supplementary Figure S3. Bootstrap edge-weight accuracy for the PSQI component network**

**Supplementary Figure S4. Case-dropping bootstrap stability of strength centrality for the PSQI component network**

**Supplementary Figure S5. Sensitivity network of global sleep quality**

**Supplementary Figure S6. Sensitivity network of sleep components**

**Supplementary Figure S7. Subgroup comparison of global sleep quality network**

**(A) global sleep quality network of ≤120 cumulative days abroad subgroup.** (B) **global sleep quality network of >120 cumulative days abroad subgroup.** Networks were estimated using the same EBICglasso procedure as in the primary analysis. Nodes represent global PSQI and psychological, occupational, environmental, and social support variables. Blue edges indicate positive associations and red edges indicate negative associations. Exploratory subgroup networks showed minor descriptive differences in several weak edges, including the PSQI–social support edge, but overall network structure and global strength did not differ significantly between the ≤120-day and >120-day groups.

**Supplementary Figure S8. Subgroup comparison of PSQI component network**

**(A) PSQI component network of ≤120 cumulative days abroad subgroup.** (B) **PSQI component network of >120 cumulative days abroad subgroup.** Networks were estimated separately for employees with (A) ≤120 cumulative days abroad and (B) >120 cumulative days abroad using the same EBICglasso procedure as in the primary analysis. Sleep nodes represent the seven PSQI components. Blue edges indicate positive associations and red edges indicate negative associations. The same node order and edge-weight scaling were used across panels.

## Supplementary Tables

**Table S1. Item-level descriptive statistics and psychometric indices for the Iraq-Specific Occupational and Environmental Stress Scale**

| **Item** | **Mean** | **SD** | **Corrected item–total correlation** | **Cronbach’s α if item deleted** | **First-component loading** |
| --- | --- | --- | --- | --- | --- |
| Local social instability and unrest | 0.485 | 0.726 | 0.730 | 0.934 | 0.788 |
| Local religion, cultural norms, and taboos | 0.331 | 0.665 | 0.749 | 0.933 | 0.802 |
| Exposure to armed groups and military equipment | 0.552 | 0.799 | 0.780 | 0.931 | 0.831 |
| Frequent checkpoint inspections during travel | 0.610 | 0.857 | 0.787 | 0.931 | 0.832 |
| Protests, demonstrations, roadblocks, or strikes around the oilfield | 0.478 | 0.763 | 0.784 | 0.931 | 0.835 |
| News exposure about unrest or violent incidents within Iraq | 0.482 | 0.725 | 0.820 | 0.930 | 0.864 |
| Fieldwork-related travel within Iraq | 0.421 | 0.720 | 0.775 | 0.932 | 0.824 |
| Harsh climate, geography, or wildlife | 0.628 | 0.884 | 0.733 | 0.934 | 0.783 |
| Odors, noise, dust, and other adverse conditions within the oilfield | 0.527 | 0.772 | 0.733 | 0.934 | 0.782 |
| Workplace friction in cross-cultural settings | 0.361 | 0.690 | 0.661 | 0.937 | 0.718 |

**Note.** N = 826. Items were rated on a 5-point Likert-type scale from 0 (no stress) to 4 (extreme stress, work nearly impossible). The IOESS-10 score was calculated as the mean of all items, with the corresponding total score ranging from 0 to 40. Internal consistency was satisfactory (Cronbach’s α = 0.939; McDonald’s ω = 0.949). The Kaiser–Meyer–Olkin value was 0.940, and Bartlett’s test of sphericity was significant, χ²(45) = 6129.28, p < 0.001. Principal component analysis of the item correlation matrix supported a dominant first component, with a first eigenvalue of 6.508 and 65.1% of variance explained. The total score was 4.875 ± 6.132, and the mean item score was 0.488 ± 0.613. A floor score of 0 was observed in 36.7% of participants, whereas no participant reached the ceiling score of 40.

**Table S2. Item-level descriptive statistics and psychometric indices for the Social Support Questionnaire**

| **Item** | **Mean** | **SD** | **Corrected item–total correlation** | **Cronbach’s α if item deleted** | **First-component loading** |
| --- | --- | --- | --- | --- | --- |
| Instrumental support from supervisors | 3.547 | 0.699 | 0.636 | 0.903 | 0.709 |
| Emotional support from supervisors | 3.429 | 0.776 | 0.717 | 0.897 | 0.781 |
| Instrumental support from coworkers | 3.573 | 0.638 | 0.761 | 0.892 | 0.818 |
| Emotional support from coworkers | 3.525 | 0.673 | 0.768 | 0.891 | 0.827 |
| Instrumental support from spouse/family members | 3.749 | 0.567 | 0.672 | 0.900 | 0.763 |
| Emotional support from spouse/family members | 3.770 | 0.533 | 0.638 | 0.903 | 0.733 |
| Instrumental support from friends | 3.558 | 0.660 | 0.746 | 0.893 | 0.824 |
| Emotional support from friends | 3.573 | 0.653 | 0.736 | 0.894 | 0.817 |

**Note.** N = 826. Items were rated on a 4-point Likert-type scale from 1 (never) to 4 (always) and summed to yield a total score ranging from 8 to 32. Higher scores indicate stronger perceived social support, reflecting greater perceived availability of instrumental and emotional support from supervisors, coworkers, family members, and friends. Internal consistency was satisfactory (Cronbach’s α = 0.909; McDonald’s ω = 0.928). The Kaiser–Meyer–Olkin value was 0.802, and Bartlett’s test of sphericity was significant, χ²(28) = 5134.49, p < 0.001. Principal component analysis of the item correlation matrix supported a dominant first component, with a first eigenvalue of 4.930 and 61.6% of variance explained. The total score was 28.724 ± 4.084, and the mean item score was 3.590 ± 0.511. A floor score of 8 was observed in 0.1% of participants, whereas a ceiling score of 32 was observed in 47.2%.

**Table S3. Subgroup replication of psychometric properties by company**

| **Scale** | **Company** | **N** | **Cronbach’s α** | **First-component loading range** | **Corrected item–total correlation range** | **Floor/Ceiling (%)** |
| --- | --- | --- | --- | --- | --- | --- |
| IOESS-10 | COSL | 380 | 0.942 | 0.771–0.862 | 0.716–0.822 | 32.4/0.0 |
| IOESS-10 | CSCEC | 340 | 0.941 | 0.690–0.892 | 0.636–0.852 | 45.0/0.0 |
| IOESS-10 | AO | 106 | 0.918 | 0.556–0.851 | 0.488–0.798 | 25.5/0.0 |
| Social Support Questionnaire | COSL | 380 | 0.897 | 0.688–0.824 | 0.600–0.747 | 0.0/45.8 |
| Social Support Questionnaire | CSCEC | 340 | 0.919 | 0.709–0.858 | 0.647–0.796 | 0.3/48.5 |
| Social Support Questionnaire | AO | 106 | 0.898 | 0.668–0.823 | 0.577–0.752 | 0.0/48.1 |

Note. IOESS-10 = Iraq-Specific Occupational and Environmental Stress Scale. KMO = Kaiser–Meyer–Olkin measure of sampling adequacy. COSL = China Oilfield Services Limited; CSCEC = China State Construction Engineering Corporation; AO = Anton Oilfield Services Principal component analysis was conducted within each company subgroup. Across company subgroups, KMO values ranged from 0.873 to 0.931 for the IOESS-10 and from 0.733 to 0.842 for the Social Support Questionnaire; Bartlett’s tests of sphericity were significant in all subgroups, all p < 0.001. The first component explained 57.7%–66.3% of item variance for the IOESS-10 and 58.5%–65.1% for the Social Support Questionnaire. Floor and ceiling effects indicate the percentage of participants achieving the minimum or maximum possible total score.

**Table S4. Correlations of the IOESS-10 and Social Support Questionnaire with established psychosocial scales**

| **Established measure** | **IOESS-10** | | **Social support** | |
| --- | --- | --- | --- | --- |
|  | **r** | **p** | **r** | **p** |
| Occupational stress (COSS-17) | 0.434 | <0.001 | -0.533 | <0.001 |
| Depressive symptoms (PHQ-9) | 0.600 | <0.001 | -0.472 | <0.001 |
| Anxiety symptoms (GAD-7) | 0.577 | <0.001 | -0.470 | <0.001 |
| Emotional exhaustion | 0.597 | <0.001 | -0.476 | <0.001 |
| Cynicism | 0.552 | <0.001 | -0.453 | <0.001 |
| Reduced professional efficacy | 0.176 | <0.001 | -0.299 | <0.001 |
| PSQI global score | 0.559 | <0.001 | -0.409 | <0.001 |

**Note.** IOESS-10 = Iraq-Specific Occupational and Environmental Stress Scale; PSQI = Pittsburgh Sleep Quality Index. r refers to Spearman’s rank correlation coefficient, with positive values indicating positive associations and negative values indicating inverse associations. Higher scores on the social support questionnaire indicate stronger perceived social support. Reduced professional efficacy was reverse-scored, with higher scores indicating lower professional efficacy.

**Table S5. Multicollinearity diagnostics for Multiple linear regression Model 3**

| Variable | GVIF | Df | GVIF^1/(2*Df)^ |
| --- | --- | --- | --- |
| Age | 1.951 | 1 | 1.397 |
| Education | 3.801 | 2 | 1.396 |
| Marriage | 1.573 | 1 | 1.254 |
| Children | 2.194 | 2 | 1.217 |
| smoking | 1.207 | 1 | 1.098 |
| drinking | 1.187 | 1 | 1.089 |
| Exercise | 1.445 | 2 | 1.096 |
| Roommate | 3.338 | 2 | 1.352 |
| Company | 6.364 | 2 | 1.588 |
| Job | 2.309 | 2 | 1.233 |
| Years Of Working | 1.829 | 3 | 1.106 |
| Cumulative days abroad | 1.896 | 3 | 1.112 |
| Overtime per week | 1.369 | 2 | 1.082 |
| Shift | 1.704 | 2 | 1.143 |
| PHQ-9 | 3.681 | 1 | 1.919 |
| GAD-7 | 3.499 | 1 | 1.870 |
| COSS1-7 | 2.052 | 1 | 1.432 |
| IOESS | 1.805 | 1 | 1.344 |
| Social support | 1.566 | 1 | 1.251 |
| Emotional exhaustion | 3.558 | 1 | 1.886 |
| Cynicism | 2.899 | 1 | 1.703 |
| Reduced professional efficacy | 1.258 | 1 | 1.121 |

**Note.** GVIF = generalized variance inflation factor; Df = degrees of freedom. For multi-level categorical predictors, the adjusted GVIF (GVIF^^1/(2·Df)^) is reported to place terms with different Df on a comparable scale; higher values indicate greater multicollinearity.

**Table S6. edge list of global sleep disturbance network**

| **Node1** | **Node2** | **Weight** | **Sign** |
| --- | --- | --- | --- |
| Anxiety symptoms | Depressive symptoms | 0.440 | positive |
| Cynicism | Emotional exhaustion | 0.410 | positive |
| Occupational stress | Social support | -0.280 | negative |
| Depressive symptoms | Emotional exhaustion | 0.250 | positive |
| Depressive symptoms | Global sleep quality | 0.240 | positive |
| Occupational stress | Reduced professional efficacy | 0.190 | positive |
| Environmental stress | Global sleep quality | 0.180 | positive |
| Emotional exhaustion | Occupational stress | 0.160 | positive |
| Environmental stress | Social support | -0.140 | negative |
| Anxiety symptoms | Emotional exhaustion | 0.130 | positive |
| Emotional exhaustion | Global sleep quality | 0.130 | positive |
| Anxiety symptoms | Cynicism | 0.120 | positive |
| Reduced professional efficacy | Social support | -0.110 | negative |
| Anxiety symptoms | Global sleep quality | 0.100 | positive |
| Cynicism | Environmental stress | 0.100 | positive |
| Depressive symptoms | Environmental stress | 0.090 | positive |
| Emotional exhaustion | Environmental stress | 0.090 | positive |
| Cynicism | Occupational stress | 0.090 | positive |
| Anxiety symptoms | Environmental stress | 0.080 | positive |
| Anxiety symptoms | Social support | -0.060 | negative |
| Cynicism | Depressive symptoms | 0.060 | positive |
| Cynicism | Social support | -0.050 | negative |
| Cynicism | Reduced professional efficacy | 0.040 | positive |
| Global sleep quality | Social support | -0.040 | negative |
| Anxiety symptoms | Occupational stress | 0.040 | positive |
| Depressive symptoms | Social support | -0.040 | negative |
| Environmental stress | Occupational stress | 0.030 | positive |
| Depressive symptoms | Occupational stress | 0.020 | positive |
| Global sleep quality | Occupational stress | 0.010 | positive |
| Emotional exhaustion | Social support | -0.010 | negative |

**Note:** The table presents the edge list of the global sleep disturbance network. Edge weights are regularized partial correlation coefficients estimated using EBICglasso based on Spearman correlation matrices. Positive values indicate positive associations and negative values indicate negative associations after conditioning on all other variables in the network. Only nonzero edges are shown.

**Table S7. Centrality indices of global sleep quality network**

| **Node** | **Betweenness** | **Closeness** | **Strength** | **Expected influence** |
| --- | --- | --- | --- | --- |
| Global sleep quality | 2 | 0.012 | 0.692 | 0.612 |
| Depressive symptoms | 5 | 0.015 | 1.142 | 1.068 |
| Anxiety symptoms | 0 | 0.012 | 0.978 | 0.849 |
| Occupational stress | 10 | 0.014 | 0.813 | 0.260 |
| Emotional exhaustion | 12 | 0.017 | 1.185 | 1.172 |
| Cynicism | 0 | 0.014 | 0.872 | 0.778 |
| Reduced professional efficacy | 0 | 0.009 | 0.339 | 0.118 |
| Environmental stress | 1 | 0.012 | 0.717 | 0.431 |
| Social support | 2 | 0.012 | 0.725 | -0.725 |

**Note:** The table presents node centrality indices for the global sleep disturbance network. Strength is defined as the sum of the absolute weights of all edges connected to a node. Expected influence is defined as the sum of all edge weights connected to a node while retaining the sign of the associations. Higher values indicate a more central position in the estimated network.

**Table S8. Edge list of PSQI components network**

| **Node1** | **Node2** | **Weight** | **Sign** |
| --- | --- | --- | --- |
| Anxiety symptoms | Depressive symptoms | 0.427 | positive |
| Cynicism | Emotional exhaustion | 0.400 | positive |
| Occupational stress | Social support | -0.274 | negative |
| Depressive symptoms | Emotional exhaustion | 0.244 | positive |
| Sleep duration | Subjective sleep quality | 0.244 | positive |
| Sleep latency | Subjective sleep quality | 0.225 | positive |
| Habitual sleep efficiency | Sleep duration | 0.221 | positive |
| Environmental stress | Sleep disturbances | 0.191 | positive |
| Occupational stress | Reduced professional efficacy | 0.189 | positive |
| Daytime dysfunction | Subjective sleep quality | 0.187 | positive |
| Daytime dysfunction | Emotional exhaustion | 0.170 | positive |
| Emotional exhaustion | Occupational stress | 0.158 | positive |
| Daytime dysfunction | Sleep disturbances | 0.144 | positive |
| Environmental stress | Social support | -0.138 | negative |
| Anxiety symptoms | Emotional exhaustion | 0.119 | positive |
| Sleep disturbances | Sleep latency | 0.115 | positive |
| Anxiety symptoms | Cynicism | 0.114 | positive |
| Reduced professional efficacy | Social support | -0.113 | negative |
| Daytime dysfunction | Sleep duration | 0.109 | positive |
| Anxiety symptoms | Daytime dysfunction | 0.107 | positive |
| Sleep disturbances | Subjective sleep quality | 0.101 | positive |
| Depressive symptoms | Sleep latency | 0.097 | positive |
| Cynicism | Environmental stress | 0.093 | positive |
| Emotional exhaustion | Environmental stress | 0.089 | positive |
| Daytime dysfunction | Depressive symptoms | 0.089 | positive |
| Cynicism | Occupational stress | 0.088 | positive |
| Depressive symptoms | Environmental stress | 0.087 | positive |
| Sleep duration | Sleep latency | 0.081 | positive |
| Depressive symptoms | Sleep disturbances | 0.080 | positive |
| Anxiety symptoms | Environmental stress | 0.070 | positive |
| Reduced professional efficacy | Sleep duration | -0.066 | negative |
| Anxiety symptoms | Social support | -0.065 | negative |
| Depressive symptoms | Subjective sleep quality | 0.060 | positive |
| Depressive symptoms | Sleep duration | 0.058 | positive |
| Cynicism | Depressive symptoms | 0.052 | positive |
| Habitual sleep efficiency | Sleep latency | 0.051 | positive |
| Cynicism | Reduced professional efficacy | 0.047 | positive |
| Sleep disturbances | Sleep duration | 0.047 | positive |
| Cynicism | Daytime dysfunction | 0.046 | positive |
| Cynicism | Social support | -0.046 | negative |
| Environmental stress | Subjective sleep quality | 0.045 | positive |
| Sleep medication use | Subjective sleep quality | 0.040 | positive |
| Daytime dysfunction | Environmental stress | 0.039 | positive |
| Depressive symptoms | Social support | -0.039 | negative |
| Environmental stress | Sleep medication use | 0.038 | positive |
| Anxiety symptoms | Occupational stress | 0.035 | positive |
| Sleep disturbances | Social support | -0.034 | negative |
| Anxiety symptoms | Sleep disturbances | 0.034 | positive |
| Anxiety symptoms | Subjective sleep quality | 0.034 | positive |
| Occupational stress | Sleep latency | 0.030 | positive |
| Environmental stress | Sleep duration | 0.029 | positive |
| Occupational stress | Sleep medication use | 0.029 | positive |
| Environmental stress | Occupational stress | 0.028 | positive |
| Anxiety symptoms | Sleep latency | 0.027 | positive |
| Daytime dysfunction | Sleep medication use | 0.027 | positive |
| Depressive symptoms | Occupational stress | 0.019 | positive |
| Emotional exhaustion | Sleep disturbances | 0.014 | positive |
| Sleep medication use | Social support | -0.012 | negative |
| Social support | Subjective sleep quality | -0.012 | negative |
| Habitual sleep efficiency | Reduced professional efficacy | 0.011 | positive |
| Habitual sleep efficiency | Occupational stress | 0.009 | positive |
| Emotional exhaustion | Social support | -0.009 | negative |
| Emotional exhaustion | Subjective sleep quality | 0.009 | positive |
| Emotional exhaustion | Sleep duration | 0.008 | positive |
| Occupational stress | Subjective sleep quality | 0.008 | positive |
| Depressive symptoms | Reduced professional efficacy | 0.007 | positive |
| Reduced professional efficacy | Subjective sleep quality | 0.007 | positive |
| Emotional exhaustion | Sleep medication use | 0.002 | positive |

**Note:** The table presents the edge list of the component-level sleep network including the seven PSQI domains and psychosocial variables. Edge weights are regularized partial correlation coefficients estimated using EBICglasso based on Spearman correlation matrices. Positive values indicate positive associations and negative values indicate negative associations after conditioning on all other variables in the network. Only nonzero edges are shown.

**Table S9. Centrality indices** **of PSQI components network**

| **Node** | **Betweenness** | **Closeness** | **Strength** | **Expected influence** |
| --- | --- | --- | --- | --- |
| Subjective sleep quality | 14 | 0.005 | 0.971 | 0.947 |
| Sleep latency | 1 | 0.005 | 0.626 | 0.626 |
| Sleep duration | 15 | 0.005 | 0.863 | 0.731 |
| Habitual sleep efficiency | 0 | 0.004 | 0.293 | 0.293 |
| Sleep disturbances | 6 | 0.005 | 0.759 | 0.691 |
| Sleep medication use | 0 | 0.002 | 0.147 | 0.124 |
| Daytime dysfunction | 15 | 0.006 | 0.917 | 0.917 |
| Depressive symptoms | 9 | 0.005 | 1.259 | 1.180 |
| Anxiety symptoms | 0 | 0.005 | 1.031 | 0.901 |
| Occupational stress | 11 | 0.005 | 0.868 | 0.319 |
| Emotional exhaustion | 22 | 0.006 | 1.223 | 1.205 |
| Cynicism | 0 | 0.005 | 0.886 | 0.795 |
| Reduced professional efficacy | 4 | 0.004 | 0.440 | 0.083 |
| Environmental stress | 8 | 0.005 | 0.847 | 0.570 |
| Social support | 4 | 0.004 | 0.741 | -0.741 |

**Note:** The table presents node centrality indices for the component-level sleep network. Strength is defined as the sum of the absolute weights of all edges connected to a node. Expected influence is defined as the sum of all edge weights connected to a node while retaining the sign of the associations. Higher values indicate a more central position in the estimated network.

**Table S10. Node redundancy analysis for the global PSQI and PSQI component networks.**

| **Network** | **Suggested redundant pair** | **Proportion of significantly different correlations** |
| --- | --- | --- |
| Global Sleep quality network | PSQI – environmental stress | 0.143 |
| PSQI component network | No suggested reductions | — |

**Note.** Suggested reductions were identified when less than 25% of correlations with other nodes significantly differed between a pair of nodes.

**Table S11. Summary of ggmModSelect sensitivity analyses**

| **Network** | **EBICglasso edges** | **ggmModSelect edges** | **Retained edges** | **Edge retention rate** | **Same direction among retained edges** | **Edge-weight Spearman rho** | **Strength-rank Spearman rho** | **Expected influence-rank Spearman rho** |
| --- | --- | --- | --- | --- | --- | --- | --- | --- |
| Global PSQI network | 30 | 17 | 17 | 56.7% | 100.0% | 0.895 | 1.000 | 0.983 |
| PSQI component network | 68 | 29 | 29 | 42.6% | 100.0% | 0.770 | 0.957 | 0.968 |

**Table S12. Edge-level comparison between EBICglasso and ggmModSelect networks**

| **Network** | **Node 1** | **Node 2** | **EBICglasso weight** | **ggmModSelect weight** |
| --- | --- | --- | --- | --- |
| **Global PSQI network** | Depressive symptoms | Anxiety symptoms | 0.440 | 0.499 |
|  | Emotional exhaustion | Cynicism | 0.415 | 0.440 |
|  | Occupational stress | Social support | -0.277 | -0.306 |
|  | Depressive symptoms | Emotional exhaustion | 0.251 | 0.265 |
|  | Global PSQI score | Depressive symptoms | 0.237 | 0.278 |
|  | Occupational stress | Reduced professional efficacy | 0.187 | 0.216 |
|  | Global PSQI score | Environmental stress | 0.179 | 0.216 |
|  | Occupational stress | Emotional exhaustion | 0.160 | 0.197 |
|  | Environmental stress | Social support | -0.143 | -0.197 |
|  | Anxiety symptoms | Emotional exhaustion | 0.135 | 0.143 |
|  | Global PSQI score | Emotional exhaustion | 0.125 | 0.158 |
|  | Anxiety symptoms | Cynicism | 0.121 | 0.144 |
|  | Reduced professional efficacy | Social support | -0.110 | -0.131 |
|  | Global PSQI score | Anxiety symptoms | 0.102 | 0.000 |
|  | Cynicism | Environmental stress | 0.099 | 0.165 |
|  | Depressive symptoms | Environmental stress | 0.094 | 0.157 |
|  | Emotional exhaustion | Environmental stress | 0.093 | 0.000 |
|  | Occupational stress | Cynicism | 0.089 | 0.116 |
|  | Anxiety symptoms | Environmental stress | 0.078 | 0.000 |
|  | Anxiety symptoms | Social support | -0.065 | -0.130 |
|  | Depressive symptoms | Cynicism | 0.059 | 0.000 |
|  | Cynicism | Social support | -0.047 | 0.000 |
|  | Cynicism | Reduced professional efficacy | 0.041 | 0.000 |
|  | Global PSQI score | Social support | -0.040 | 0.000 |
|  | Anxiety symptoms | Occupational stress | 0.037 | 0.000 |
|  | Depressive symptoms | Social support | -0.037 | 0.000 |
|  | Occupational stress | Environmental stress | 0.030 | 0.000 |
|  | Depressive symptoms | Occupational stress | 0.024 | 0.000 |
|  | Global PSQI score | Occupational stress | 0.009 | 0.000 |
|  | Emotional exhaustion | Social support | -0.006 | 0.000 |
| **PSQI component network** | Depressive symptoms | Anxiety symptoms | 0.427 | 0.499 |
|  | Emotional exhaustion | Cynicism | 0.400 | 0.438 |
|  | Occupational stress | Social support | -0.274 | -0.307 |
|  | Depressive symptoms | Emotional exhaustion | 0.244 | 0.325 |
|  | Subjective sleep quality | Sleep duration | 0.244 | 0.288 |
|  | Subjective sleep quality | Sleep latency | 0.225 | 0.274 |
|  | Sleep duration | Habitual sleep efficiency | 0.221 | 0.263 |
|  | Sleep disturbances | Environmental stress | 0.191 | 0.216 |
|  | Occupational stress | Reduced professional efficacy | 0.189 | 0.216 |
|  | Subjective sleep quality | Daytime dysfunction | 0.187 | 0.225 |
|  | Daytime dysfunction | Emotional exhaustion | 0.170 | 0.231 |
|  | Occupational stress | Emotional exhaustion | 0.158 | 0.201 |
|  | Sleep disturbances | Daytime dysfunction | 0.144 | 0.171 |
|  | Environmental stress | Social support | -0.138 | -0.191 |
|  | Anxiety symptoms | Emotional exhaustion | 0.119 | 0.000 |
|  | Sleep latency | Sleep disturbances | 0.115 | 0.121 |
|  | Anxiety symptoms | Cynicism | 0.114 | 0.171 |
|  | Reduced professional efficacy | Social support | -0.113 | -0.131 |
|  | Sleep duration | Daytime dysfunction | 0.109 | 0.114 |
|  | Daytime dysfunction | Anxiety symptoms | 0.107 | 0.181 |
|  | Subjective sleep quality | Sleep disturbances | 0.101 | 0.119 |
|  | Sleep latency | Depressive symptoms | 0.097 | 0.137 |
|  | Cynicism | Environmental stress | 0.093 | 0.158 |
|  | Emotional exhaustion | Environmental stress | 0.089 | 0.000 |
|  | Daytime dysfunction | Depressive symptoms | 0.089 | 0.000 |
|  | Occupational stress | Cynicism | 0.088 | 0.111 |
|  | Depressive symptoms | Environmental stress | 0.087 | 0.155 |
|  | Sleep latency | Sleep duration | 0.081 | 0.000 |
|  | Sleep disturbances | Depressive symptoms | 0.080 | 0.109 |
|  | Anxiety symptoms | Environmental stress | 0.070 | 0.000 |
|  | Sleep duration | Reduced professional efficacy | -0.066 | 0.000 |
|  | Anxiety symptoms | Social support | -0.065 | -0.132 |
|  | Subjective sleep quality | Depressive symptoms | 0.060 | 0.000 |
|  | Sleep duration | Depressive symptoms | 0.058 | 0.098 |
|  | Depressive symptoms | Cynicism | 0.052 | 0.000 |
|  | Sleep latency | Habitual sleep efficiency | 0.051 | 0.000 |
|  | Cynicism | Reduced professional efficacy | 0.047 | 0.000 |
|  | Sleep duration | Sleep disturbances | 0.047 | 0.000 |
|  | Daytime dysfunction | Cynicism | 0.046 | 0.000 |
|  | Cynicism | Social support | -0.046 | 0.000 |
|  | Subjective sleep quality | Environmental stress | 0.045 | 0.101 |
|  | Subjective sleep quality | Sleep medication use | 0.040 | 0.000 |
|  | Daytime dysfunction | Environmental stress | 0.039 | 0.000 |
|  | Depressive symptoms | Social support | -0.039 | 0.000 |
|  | Sleep medication use | Environmental stress | 0.038 | 0.106 |
|  | Anxiety symptoms | Occupational stress | 0.035 | 0.000 |
|  | Sleep disturbances | Social support | -0.034 | 0.000 |
|  | Sleep disturbances | Anxiety symptoms | 0.034 | 0.000 |
|  | Subjective sleep quality | Anxiety symptoms | 0.034 | 0.000 |
|  | Sleep latency | Occupational stress | 0.030 | 0.000 |
|  | Sleep duration | Environmental stress | 0.029 | 0.000 |
|  | Sleep medication use | Occupational stress | 0.029 | 0.000 |
|  | Occupational stress | Environmental stress | 0.028 | 0.000 |
|  | Sleep latency | Anxiety symptoms | 0.027 | 0.000 |
|  | Sleep medication use | Daytime dysfunction | 0.027 | 0.000 |
|  | Depressive symptoms | Occupational stress | 0.019 | 0.000 |
|  | Sleep disturbances | Emotional exhaustion | 0.014 | 0.000 |
|  | Sleep medication use | Social support | -0.012 | 0.000 |
|  | Subjective sleep quality | Social support | -0.012 | 0.000 |
|  | Habitual sleep efficiency | Reduced professional efficacy | 0.011 | 0.000 |
|  | Habitual sleep efficiency | Occupational stress | 0.009 | 0.000 |
|  | Emotional exhaustion | Social support | -0.009 | 0.000 |
|  | Subjective sleep quality | Emotional exhaustion | 0.009 | 0.000 |
|  | Sleep duration | Emotional exhaustion | 0.008 | 0.000 |
|  | Subjective sleep quality | Occupational stress | 0.008 | 0.000 |
|  | Depressive symptoms | Reduced professional efficacy | 0.007 | 0.000 |
|  | Subjective sleep quality | Reduced professional efficacy | 0.007 | 0.000 |
|  | Sleep medication use | Emotional exhaustion | 0.002 | 0.000 |

Note. This table includes edges that were present in at least one of the two estimators. EBICglasso was the primary network estimator. ggmModSelect was the sensitivity estimator. No new ggmModSelect edges were observed in either network.

**Table S13. Centrality comparison between EBICglasso and ggmModSelect networks**

| **Network** | **Node** | **strength** | | **strength rank** | | **expected influence** | | **EI rank** | |
| --- | --- | --- | --- | --- | --- | --- | --- | --- | --- |
|  |  | **EBICglasso** | **ggmModSelect** | **EBICglasso** | **ggmModSelect** | **EBICglasso** | **ggmModSelect** | **EBICglasso** | **ggmModSelect** |
| Global PSQI network | Emotional exhaustion | 1.185 | 1.203 | 1 | 1 | 1.172 | 1.203 | 1 | 1 |
|  | Depressive symptoms | 1.142 | 1.199 | 2 | 2 | 1.068 | 1.199 | 2 | 2 |
|  | Anxiety symptoms | 0.978 | 0.916 | 3 | 3 | 0.849 | 0.656 | 3 | 4 |
|  | Cynicism | 0.872 | 0.867 | 4 | 4 | 0.778 | 0.867 | 4 | 3 |
|  | Occupational stress | 0.813 | 0.836 | 5 | 5 | 0.260 | 0.224 | 7 | 7 |
|  | Social support | 0.725 | 0.763 | 6 | 6 | -0.725 | -0.763 | 9 | 9 |
|  | Environmental stress | 0.717 | 0.735 | 7 | 7 | 0.431 | 0.342 | 6 | 6 |
|  | Global PSQI score | 0.692 | 0.651 | 8 | 8 | 0.612 | 0.651 | 5 | 5 |
|  | Reduced professional efficacy | 0.339 | 0.347 | 9 | 9 | 0.118 | 0.086 | 8 | 8 |
| PSQI component network | Depressive symptoms | 1.259 | 1.323 | 1 | 1 | 1.180 | 1.323 | 2 | 1 |
|  | Emotional exhaustion | 1.223 | 1.195 | 2 | 2 | 1.205 | 1.195 | 1 | 2 |
|  | Subjective sleep quality | 0.971 | 1.007 | 4 | 3 | 0.947 | 1.007 | 3 | 3 |
|  | Anxiety symptoms | 1.031 | 0.983 | 3 | 4 | 0.901 | 0.720 | 5 | 8 |
|  | Environmental stress | 0.847 | 0.926 | 9 | 5 | 0.570 | 0.544 | 10 | 9 |
|  | Daytime dysfunction | 0.917 | 0.922 | 5 | 6 | 0.917 | 0.922 | 4 | 4 |
|  | Cynicism | 0.886 | 0.878 | 6 | 7 | 0.795 | 0.878 | 6 | 5 |
|  | Occupational stress | 0.868 | 0.835 | 7 | 8 | 0.319 | 0.220 | 11 | 12 |
|  | Sleep duration | 0.863 | 0.764 | 8 | 9 | 0.731 | 0.764 | 7 | 6 |
|  | Social support | 0.741 | 0.761 | 11 | 10 | -0.741 | -0.761 | 15 | 15 |
|  | Sleep disturbances | 0.759 | 0.736 | 10 | 11 | 0.691 | 0.736 | 8 | 7 |
|  | Sleep latency | 0.626 | 0.533 | 12 | 12 | 0.626 | 0.533 | 9 | 10 |
|  | Reduced professional efficacy | 0.440 | 0.347 | 13 | 13 | 0.083 | 0.086 | 14 | 14 |
|  | Habitual sleep efficiency | 0.293 | 0.263 | 14 | 14 | 0.293 | 0.263 | 12 | 11 |
|  | Sleep medication use | 0.147 | 0.106 | 15 | 15 | 0.124 | 0.106 | 13 | 13 |

Note. Strength is the sum of absolute edge weights connected to a node. Expected influence is the sum of signed edge weights connected to a node
